# Clinical Features and Visual Outcomes of Optic Neuritis in Chinese Children

**DOI:** 10.1155/2016/9167361

**Published:** 2016-09-20

**Authors:** Huanfen Zhou, Wei Wang, Quangang Xu, Shaoying Tan, Shuo Zhao, Mo Yang, Chunxia Peng, Shihui Wei

**Affiliations:** ^1^Department of Ophthalmology, The First Affiliated Hospital of Chinese People's Liberation Army General Hospital, Beijing, China; ^2^Department of Ophthalmology, Chinese PLA General Hospital, Beijing, China; ^3^Zhongshan Ophthalmic Center, State Key Laboratory of Ophthalmology, Sun Yat-sen University, Guangzhou, China; ^4^Department of Neurology, Chinese PLA General Hospital, Beijing, China; ^5^Department of Ophthalmology, Beijing Hospital, Beijing, China

## Abstract

*Purpose*. Although optic neuritis (ON) in children is relatively common, visual outcomes and factors associated with the condition have not been well documented. The aim of this study was to evaluate the clinical features and visual outcomes of ON in Chinese children.* Methods*. Patients with a first episode of ON at a tertiary neuroophthalmic centre in China were assessed and followed up for at least three months. Visual outcomes and clinical, laboratory, and neuroimaging findings were reviewed. In patients with bilateral ON, only the eyes with worse visual acuity (VA) at presentation were used for statistical analysis.* Results*. Seventy-six children (76 eyes) with a first episode of ON were included. The mean age was 11.8 years, 60.5% were females, and 48.7% had bilateral involvement. The children were followed up for an average of 18.5 months (age range, 3–48 months). Vision loss at presentation was severe, with VA < 20/200 in 37 eyes (48.7%). At the final visit, 3 (3.9%) eyes had VA of at least 20/20, and 41 (53.9%) eyes had VA of at least 20/40. The final VA in 35 eyes (46.1%) was worse than 20/40. Children aged ≤ 10 years had better predicted visual outcomes when compared to children over 10 years (odds ratio = 2.73, 95% confidential interval: 1.05–7.07, and *P* = 0.039). The other features of this cohort, such as sex, experienced bilateral attack, VA at presentation, presence of optic disc edema, systemic diseases, magnetic resonance imaging (MRI) findings, and aquaporin-4 (AQP-4) antibody status, were not significantly correlated with the final visual outcome.* Conclusion*. The data revealed the clinical characteristics and visual outcomes of ON in Chinese children. ON in children was associated with severe vision loss and relatively good visual recovery. The age at onset could predict the final visual function.

## 1. Introduction

Optic neuritis (ON) is an inflammatory process of the optic nerve, which may be isolated or closely correlated with systemic demyelinating diseases such as multiple sclerosis (MS) and neuromyelitis optica spectrum disorders (NMOSDs) [1, 2]. Aquaporin-4 (AQP-4) antibody testing has been added to the diagnostic assessment for ON and has prognostic value [3, 4]. The prevalence of ON in children is lower than that in adults. Furthermore, ON in children is distinguished by greater bilateral involvement, higher prevalence of optic disc edema (ODE), and lower conversion into MS [5]. In the optic neuritis treatment trial (ONTT), 50% of adults with ON, 72% patients with one or more lesions in brain MRI, and 25% of patients without lesion in brain MRI progressed to MS during the 15-year follow-up [6, 7]. With regard to children with ON, a meta-analysis demonstrated that only 19% of the children developed MS after an average follow-up of 6.3 years [8].

The influence of race/ethnicity on features and outcomes of adults with ON has been well documented. In ONTT, people of African American origin were found to have lower visual acuity (VA) and contrast sensitivity at onset and faster vision recovery but worse final visual outcomes in comparison with white patients with ON [9]. Epidemiological studies also showed that African Americans had more severe vision loss, worse disability scores, more retinal atrophy, and accelerated retinal nerve fibre-layer thinning compared to Caucasian Americans [10–12]. Furthermore, MS is rare in East Asian populations such as Chinese, Korean, and Japanese populations, and the prognosis of ON in these populations differs from that in American and European populations. We previously demonstrated a higher prevalence of serum AQP-4 antibody, severe vision loss, and poor vision recovery in Chinese adults with ON, with only 26.1% of patients having VA of 20/20 or better and 39.1% of patients having VA of 20/100 or worse at the final visit [13, 14].

Although the visual outcomes of adults with ON are well documented in the ONTT, there are few trials on the outcomes for children with ON. In 2014, Wan et al. reviewed 46 American children (46 eyes) with a first episode of ON and reported the vision in 35 of 36 eyes (97%) recovered to >20/40 after one year [15]. However, most of the children in their study were white. Studies on other races are urgently needed. Therefore, to fill the gap, we performed a retrospective study to investigate the clinical features and visual outcomes of ON in Chinese children.

## 2. Methods

This was a retrospective observational cohort study on hospitalized patients diagnosed with ON in the neuroophthalmology department of Chinese People's Liberation Army General Hospital (PLAGH). Patients were recruited between January 2012 and December 2014. The study protocol was approved by the institutional review board at the Chinese PLAGH and performed in accord with the tenets of the Helsinki Declaration. Informed consent was obtained from all the patients or their parents.

### 2.1. Patients

Patients who presented with a first episode of ON were identified by searching electronic medical records. The diagnosis of ON was confirmed by both an experienced neurologist (QX) and ophthalmologist (SW). Patients included in this study had to fulfil the following criteria: (1) the first episode of ON with best corrected visual acuity (BCVA) loss; (2) age < 18 years; (3) follow-up of ≥3 months; (4) no prior steroid treatment; and (5) objective evidence of visual abnormalities: relative afferent papillary defect (RAPD), visual field defects, and abnormal visual evoke potential (VEP). Patients with any evidence of the following conditions were excluded: (1) uncertainty in the diagnosis; (2) other types of optic neuropathy such as compressive, hereditary, vascular, toxic, traumatic, metabolic, or infiltrative optic neuropathy; (3) other ocular diseases such as amblyopia, uveitis, or glaucoma; (4) a previous episode of ON; and (5) systemic diseases, such as epilepsy and malignancy. Patients diagnosed with MS, NMOSD, acute disseminated encephalomyelitis (ADEM), or systemic lupus erythematosus (SLE) were not excluded.

### 2.2. Study Outcomes

The following data were extracted from the medical records using a standardized form: age, sex, laterality, ocular symptoms, pupil function test, fundus findings, colour vision test, and laboratory findings including AQP-4 IgG status and cerebral spinal fluid (CSF) analysis, orbit magnetic resonance imaging (MRI) information, treatment, follow-up, best corrected visual acuity (VA) at presentation, and final VA. The serum AQP-4 antibody examination was assessed using the cell-based assay described previously by our group [31]. The VA in all the children was evaluated using the Snellen visual chart, and the data were transformed as the logarithm of the minimum angle of resolution (log⁡MAR) values [16]. The MRI images were reviewed by the same experienced radiologist. Moreover, MS and NMOSD were diagnosed according to the latest international criteria [17, 32]. In patients with bilateral ON, only the eyes with worse BCVA at presentation were included in this study.

### 2.3. Statistical Analyses

Only one eye of each patient was used for statistical analysis. In patients with bilateral ON, only the eyes with worse visual acuity (VA) at presentation were used for statistical analysis to exclude intereye correlations. All statistical analyses were performed using SPSS 20.0 software (IBM Corporation, New York, USA). Fisher's exact test was used to compare categorical variables between groups and determine the predictors of a favourable visual outcome (final VA ≥ 20/40). No adjustment for multiple comparisons was made owing to the exploratory nature of the study. In addition, logistic regression analyses were performed to calculate the odds ratio of poor visual outcome as a function of age onset, sex, laterality, VA at presentation, presence of ODE, systemic diseases, MRI findings, and AQP-4 IgG status. A value of *P* < 0.05 was considered statistically significant.

## 3. Results

### 3.1. Demographic and Clinical Characteristics

Seventy-six children were eligible for this study, with 30 male and 46 female children.  Table 1 summarized the demographic and clinical characteristics of these patients (unit of presentation = person). 39 (51.3%) patients experienced unilateral involvement and 37 patients (48.7%) experienced bilateral involvement. The mean age at onset was 11.8 years (5–17 years). The follow-up duration ranged from 3 to 48 months, with a mean of 18.5 months (median of 11 months). Eighteen patients who experienced bilateral ON had RAPD. Of the 47 patients who underwent AQP-4 IgG testing, 13 (27.7%) were seropositive and 34 (72.3%) were seronegative. Of the 42 patients who underwent orbit MRI imaging, 35 (83.33%) had optic nerve T2 lesions and 7 (16.67%) had normal MRI findings. One patient was diagnosed with ADEM, one patient was diagnosed with SLE, three patients were diagnosed with MS, and 13 patients were diagnosed with neuromyelitis optica spectrum disorders (NMOSDs) during the follow-up. ON was the first symptom in all children and no prior myelitis attacked in children with seropositive AQP-4 antibody. During the follow-up, none of the children with AQP-4 Ab seropositive underwent attack of myelitis.

To eliminate intereye correlations, data of only one eye in each patient was included in the statistical analysis (unit of analysis = eye). Twenty-two (28.9%) eyes had ODE. Of 55 eyes with colour vision test, 11 (22%) eyes had colour vision deficit. Vision loss at presentation was severe, with VA < 20/200 in 37 (48.7%) eyes. All patients received intravenous methylprednisolone therapy (10–30 mg/kg/day) for three days, followed by oral prednisolone (1 mg/kg/day for two weeks).

### 3.2. Visual Outcomes

VA was determined in the affected eyes in unilateral ON and in the worse eyes at presentation in bilateral ON. The VA at presentation of the patients aged ≤ 10 years was not significantly different from that of the patients aged > 10 years (Table 2). 13 (43.3%) patients aged ≤ 10 years and 24 (52.2%) patients aged > 10 years had VA < 20/200. At the final visit, among those aged ≤ 10 years, the VA of 21 (70%) patients had improved to at least 20/40, as compared to that of 20 (43.5%) patients aged < 10 years.

### 3.3. Predictors of Visual Outcomes

The age at onset was significantly correlated with the final VA. The children aged ≤ 10 years had better predicted visual outcomes when compared to those older than 10 years (odds ratio = 2.73, 95% confidential interval: 1.05–7.07, and *P* = 0.039). The other features of this cohort, such as sex, experienced bilateral attack, VA at presentation, presence of ODE, systemic diseases, MRI findings, and AQP-4 IgG status, were not significantly correlated with the final visual outcomes (Table 3).

## 4. Discussion

In this study, we described the clinical features and visual outcomes of ON in Chinese children. The clinical features of this cohort included female preponderance (60.5%), common bilateral involvement (48.7%), and a high proportion of ODE (28.9%), which is consistent with previous reports of ON in various ethnic groups (Table 4) [15, 18–30, 33–36]. The vision loss was severe with 48.7% of patients having VA < 20/200. However, the visual recovery was favourable in the majority of children, with VA at least 20/40 in 53.9% of the patients. The only identified predictor of visual outcomes was the age at onset. To the best of our knowledge, this is the first study to report the characteristics of ON in children in Mainland China and represents the largest cohort of children with ON.

Few studies have reported the visual outcomes of ON in children to date. Brady et al. examined 25 children (39 eyes) with ON and reported that 30 of 39 (76%) eyes recovered to 20/40 or better after a follow-up of an average of 11 months [30]. Wilejto et al. reviewed 36 children (51 eyes) with ON in Canada and reported that the VA of 39 of 47 eyes (83%) recovered to ≥20/40 after a follow-up of 2.4 years [26]. In Japanese children with ON, Mizota et al. reported that 54 of 61 (95%) eyes recovered to 20/20 or better after a mean follow-up of 10.7 years [27]. A study of Korean children with ON reported that the VA recovered to ≥20/40 in 53.3%–80% of affected eyes but that it remained ≤20/200 in 7.7%–13.3% of eyes at the final visit [18, 22, 25, 28]. A study of Malaysian children revealed that the final VA of 21 of 28 (75%) eyes was ≥20/40 [21]. Among children with ON in Taiwan, Sun et al. reported that the VA of 20 of 24 (83.3%) eyes recovered to ≥20/40 after a mean follow-up of 14.01 months [23]. In the present study, the VA of 53.9% of the patients recovered to ≥20/40, which is similar to the visual outcomes reported in Korean children [25].

The ONTT reported that 50% of patients had VA of 20/20 or better and 68% of patients had VA of 20/40 or better after one year [37]. In American children with ON, Wan et al. reported that 81% of children had VA of 20/20 or better and 89% of children had VA of 20/40 or better at the one-year follow-up visit [15]. In this Chinese cohort, we also observed a favourable visual prognosis in the majority of the population, thereby indicating that children with ON have at least similar or better visual outcomes compared to those in adults.

The identification of predictors of visual outcomes is essential for individualized treatment. The ONTT study demonstrated that race significantly affected the visual prognosis, but it found no associations between age, sex, treatment, and visual outcomes in follow-up periods of 5, 10, or 15 years [6]. In this study, we found that the age at onset was significantly correlated with the visual outcome of the children. Brady et al. examined 25 children (39 eyes) with ON and reported a trend towards better final VA in patients aged < 6 years, but this did not have statistical significance [30]. A study in Taiwan reported that poor visual recovery (<20/40) in children was associated with younger age (≤10 years) but not with initial VA, sex, laterality, underlying diagnosis, and abnormalities of brain MRI [23]. However, two other studies found no association between the age at onset and final VA [15, 21]. The discrepancy may be caused by different ethnicities, small sample size, distribution of ages, various phenotypes of ON, or different follow-up times. Consistent with most previous studies, we confirmed that several baseline parameters such as sex, laterality, initial visual acuity, appearance of ODE, MRI findings, and treatment did not affect the final visual function [15, 19, 23, 33].

Severe vision loss, a high prevalence of AQP-4 IgG, and poor visual recovery were reported to be common in Chinese adults with ON [38]. We performed AQP-4 IgG testing in 47 patients, and 13 (27.7%) were seropositive. However, we did not observe any correlations between underlying diagnosis, AQP-4 IgG status, and final VA in this study. The small sample size in our subgroup analysis may explain the lack of correlations. Banwell et al. found that AQP-4 IgG was seropositive in 78% of children with recurrent NMO [39]. Absoud et al. reviewed 20 children with NMOSD in the UK and reported that the VA of 10 of 12 patients with seropositive AQP-4 IgG was <20/200 in at least one eye and the bilateral VA was <20/200 in 58% of the patients after a mean follow-up of 6.1 years [40]. Most previous studies of ON in children did not detect the serum AQP-4 IgG status. In a Korean study by Kim et al. the status of AQP-4 IgG was only available in three patients [18]. Thus, prospective studies with a large sample size and various ethnicities are required to clarify the influence of AQP-4 IgG on final visual outcomes in children with ON.

The strengths of this study included the uniform sample of children with the first episode of ON, standardized treatment, and detection of AQP-4 IgG status. On the other hand, this study also has a number of limitations. First, the nature of the retrospective design introduces selection bias, precluding the confirmation of the cause and effect relationship. Second, all the patients were derived from a tertiary neuroophthalmic centre in China. Thus, the findings of this study may not be applicable to a larger population or other ethnicities. Third, in the association analyses, there were a relatively small number of patients in several of the subgroups, thereby causing nonsignificance associations between certain factors and visual outcomes. Fourth, we were not able to construct any useful statistics about MS-like lesions or typical NMOSD lesions in brain MRI because only a very small number of patients received the brain MRI or the spine cord MRI. Furthermore, myelin oligodendrocyte glycoprotein (MOG) antibody was not available in our department, which was reliably associated with a spectrum of demyelinating disorders including relapsing paediatric demyelination [41, 42]. Fifth, the follow-up was not standardized and relatively short, and analyses of the conversion rate of MS and NMOSD were not performed. Further, Sun et al. reported a relatively low conversion rate (12.5%) of MS in children with ON in Taiwan and ADEM was reported to be the most common systemic cause [23]. In Korean children, Kim et al. showed that only two patients (7.7%) developed MS [18]. More studies with longer-term follow-up and standardized evaluations are necessary.

In summary, the clinical features of ON in Chinese children were similar to those of children in other Asian countries. Paediatric ON was associated with severe vision loss and relatively good visual recovery. The majority of this cohort (53.9%) had final VA of at least 20/40. Age at onset of <10 years predicted better visual outcomes. No other clinical parameters were significantly correlated with the final visual function.

## Figures and Tables

**Table 1:** Demographic and clinical characteristics of Chinese children with optic neuritis.

Features	Total	Percent (%)
Number of patients	76	100%
Age at onset, year		
Mean	11.80 ± 3.87	
Range	5–17	
Sex		
Male	30	39.5
Female	46	60.5
Experience	76	
Unilateral attack	39	51.3
Bilateral attack	37	48.7
Pupillary function test	76	
RAPD positive in unilateral	39	100
RAPD positive in bilateral	18	48.65
AQP-4 IgG status	47	
Seropositive	13	27.7
Seronegative	34	72.3
Orbit MRI	42	
Abnormal (T_2_ lesions)	35	83.33
Normal	7	16.67
Causes/associations	76	
ION	58	76.3
ADEM	1	1.3
MS	3	4.0
SLE	1	1.3
NMOSD	13	17.1
Funduscopy^*∗*^	76	
Optic disc edema	22	28.9
Normal	54	71.1
Colour vision test^*∗*^	55	
Deficit	11	20
Intact	33	60
Vision too poor to test	11	20
Presenting VA^*∗*^	76	
20/40 ≤ VA < 20/20	5	6.6
20/200 ≤ VA < 20/40	34	44.7
CF ≤ VA < 20/200	6	7.9
NLP ≤ VA < CF	31	40.8
Treatment		
IV methylprednisolone	76	100

RAPD, relative afferent papillary defect; AQP-4 IgG, anti-aquaporin-4 antibody IgG; ION, isolated optic neuritis; ADEM, acute disseminated encephalomyelitis; MS, multiple sclerosis; SLE, systemic lupus erythematosus; IV, intravenous; VA, best corrected visual acuity; CF, count finger; and NLP, no light perception.

^*∗*^Only one eye of each patient was included in the statistical analyses.

**Table 2:** Visual outcomes of Chinese children with optic neuritis.

Visual outcomes (worse eye at onset if bilateral)	Age ≤ 10 years		Age > 10 years	
	Initial	Final	Initial	Final
VA ≥ 20/20	0	3 (10%)	0	0
20/40 ≤ VA < 20/20	3 (10%)	18 (60%)	2 (4.4%)	20 (43.5%)
20/200 ≤ VA < 20/40	14 (46.7%)	7 (23.3%)	20 (43.5%)	22 (47.8%)
CF ≤ VA < 20/200	1 (3.3%)	0	5 (10.9%)	0
NLP ≤ VA < CF	12 (40%)	2 (6.7%)	19 (41.3%)	4 (8.7%)

Total	30	30	46	46

VA, visual acuity; CF, count finger; and NLP, no light perception.

**Table 3:** Factors for visual outcomes in Chinese children with optic neuritis.

Factors		Total eyes	Final VA ≥ 20/40 (%)	*P* value
Age at onset	Age ≤ 10 years	30	21 (70%)	**0.039**
	Age > 10 years	46	20 (43.5%)	

Sex	Female	46	24 (52.17%)	0.701
	Male	30	17 (56.7%)	

Experience	Unilateral attack	39	20 (51.3%)	0.632
	Bilateral attack	37	21 (56.8%)	

Initial VA	VA > CF	42	25 (59.5%)	0.278
	VA ≤ CF	34	16 (47.1%)	

Funduscopy	ODE	22	14 (63.6%)	0.501
	Normal	54	30 (55.6%)	

Systemic diseases	ADEM/MS/SLE/NMOSD	18	8 (44.4%)	0.454
	ION	58	32 (55.2%)	

Orbital MRI imaging	Normal	7	4 (57.1%)	0.934
	Abnormal (T_2_ lesions)	35	20 (58.8%)	

AQP-4 IgG status	Seropositive	13	5 (38.5%)	0.374
	Seronegative	34	18 (52.9%)	

VA, visual acuity; CF, count finger; ODE, optic disc edema; ION, isolated optic neuritis; ADEM, acute disseminated encephalomyelitis; MS, multiple sclerosis; SLE, systemic lupus erythematosus; AQP-4 IgG, anti-aquaporin-4 antibody IgG; and MRI, magnetic resonance imaging.

**Table 4:** Clinical features of children optic neuritis in previous published reports.

First author	Year	Location	Patient (eyes)	Female	Mean age	Bilateral	ODE	MS	NMOSD
Present		China	76 (76)	60.5%	11.8	48.7%	36.7%	3.95%	17.1%
Kim [18]	2015	Korea	26 (40)	54.0%	10.3	54.0%	77.0%	8.0%	0
Wan [15]	2014	USA	46 (46)	72.0%	12.6	41.0%	67.0%	39.0%	NR
Jayakody [19]	2014	USA	26 (38)	73.1%	4.5-19	46.0%	73.0%	7.7%	0
Shatriah [21]	2012	Malaysia	14 (28)	85.7%	11.1	100%	85.8%	14.3%	NR
Jo [22]	2011	Korea	20 (33)	85.0%	6.5	65.0%	75.8%	25.0%	NR
Sri-Udomkajorn [33]	2011	Thailand	31	65.0%	9.2	74.2%	55.0%	6.0%	6.5%
Sun [23]	2011	Taiwan	24 (38)	58.3%	10.1	58.3%	63.2%	12.5%	NR
Hwang [25]	2007	Korea	10 (15)	50.0%	7.31	NR	NR	NR	NR
Wilejto [26]	2006	Canada	36 (51)	58.0%	12.2	42.0%	67.0%	36.0%	2.8%
Mizota [27]	2004	Japan	41 (61)	56.0%	9.4	49.0%	74.0%	31.7%	NR
Hwang [28]	2002	Korea	23 (43)	43.0%	8.9	87.0%	51.0%	4.0%	NR
Morales [29]	2000	USA	15	60.0%	9.8	66.0%	64.0%	26.0%	NR
Brady [30]	1999	USA	25 (39)	52.0%	9.4	54.0%	NR	16.0%	NR
Visudhiphan [34]	1995	Thailand	22 (41)	54.5%	7.1	86.3%	48.7%	9.1%	NR
Kriss [35]	1988	UK	39	74.0%	8.6	74.0%	74.0%	15.0%	NR
Riikonen [36]	1988	Finland	21	NR	NR	62.0%	76.0%	43.0%	NR

ODE, optic disc edema; MS, multiple sclerosis; NMOSD, neuromyelitis optica spectrum disorders; and NR, not reported.
